# Data Intensive Genome Level Analysis for Identifying Novel, Non-Toxic Drug Targets for Multi Drug Resistant *Mycobacterium tuberculosis*

**DOI:** 10.1038/srep46595

**Published:** 2017-04-20

**Authors:** Divneet Kaur, Rintu Kutum, Debasis Dash, Samir K. Brahmachari

**Affiliations:** 1 CSIR- Institute of Genomics and Integrative Biology, New Delhi, India; 2 CSIR- Open Source Drug Discovery Unit, New Delhi, India; 3 Academy of Scientific and Innovative Research, New Delhi, India

## Abstract

We report the construction of a novel Systems Biology based virtual drug discovery model for the prediction of non-toxic metabolic targets in *Mycobacterium tuberculosis* (Mtb). This is based on a data-intensive genome level analysis and the principle of conservation of the evolutionarily important genes. In the 1623 sequenced Mtb strains, 890 metabolic genes identified through a systems approach in Mtb were evaluated for non-synonymous mutations. The 33 genes showed none or one variation in the entire 1623 strains, including 1084 Russian MDR strains. These invariant targets were further evaluated for their experimental and *in silico* essentiality as well as availability of their crystal structure in Protein Data Bank (PDB). Along with this, targets for the common existing antibiotics and the new Tb drug candidates were also screened for their variation across 1623 strains of Mtb for understanding the drug resistance. We propose that the reduced set of these reported targets could be a more effective starting point for medicinal chemists in generating new chemical leads. This approach has the potential of fueling the dried up Tuberculosis (Tb) drug discovery pipeline.

It is estimated that in adults, the second largest leading cause of death (after AIDS) is Tuberculosis (Tb); a disease primarily caused by the bacterium *Mycobacterium tuberculosis* (Mtb) whose latent form affects about one-third of the world’s population^1^. The treatment of the Tb infection, comprising first-line drugs, is usually for six to nine months. Lack of compliance (in spite of the Directly Observed Treatment, Short Course (DOTS) regime), has led to the emergence of the Multi Drug Resistant (MDR) and Extreme Drug Resistant (XDR) strains in the bacteria causing an increased public health concern^2^,^3^. The problem is further complicated by bacterial persistence, a phenomenon that allows non-mutant pathogens of an isogenic population to survive the impact of an antibiotic^4^,^5^,^6^. Given the current situation of drug resistance and tolerance against Mtb, urgent attention is required, in terms of the development of newer therapeutics and strategies. With this in mind (as one of its aims), the Open Source Drug Discovery (OSDD) project was initiated in 2008^7^,^8^, to facilitate the process of Tb drug discovery. In the present situation, understanding the complex biological responses or the systems biology of an organism is highly significant in order to improve and fasten the process of drug discovery, especially for Tb. Therefore, a systems-level analysis was undertaken with the integration of the ‘omics’ data (proteomics and genomics) incorporating all the experimental evidences from the literature, along with a comprehensive annotation of the Mtb genome with computer simulations^9^,^10^. This analysis was performed to identify the novel non-toxic drug targets, most of which has experimental evidences *in vitro* and *in vivo*.

From our previous analysis, critical and low concentration proteins required for the growth and survival of Mtb were identified using a novel Systems Biology Spindle Map (SBSM)^11^. The result of the *in silico* single gene knock-out analysis was carried out on a total of 890 metabolic genes and 961 metabolites involved in 1152 reactions to identify putative drug targets. The *in silico* single gene knock-out analysis on the 211 Metabolic Persister Genes (MPGs) revealed the potential drug targets which should have lower chances of developing drug tolerance.

Along with the systems level analysis, understanding the strain specific genetic differences in Mtb also becomes important, since Tb in humans originates from a variety of Mtb strains. A comparative genome analysis of 96 strains with H37Rv and H37Ra as reference strains has been performed with a construction of its pangenome architecture^12^. The latter was carried out with a view to understand the virulence and the drug resistance associated with genetic differences in strains from the same and/or different geographical origins. Sequencing of the complete genome of the various clinical isolates of Mtb from different populations has, therefore, opened new opportunities to address issues like bacterial drug resistance and persistence based on the principle of conservation and variations of the genome.

In order to determine the evolutionary importance of a protein, it is crucial to understand the regions within it that vary without a loss of function and those that remain strictly conserved/remain invariant across the entire population of an organism^13^. Genes resisting the mutations remain conserved, as the tolerance towards mutations is zero and any disruptions in its structural feature could be lethal for their survival. The emergence of resistance and persistence in the organism can thus be traced back by studying the variations of its entire genome in the different clinical isolates. Based on this existing knowledge, we propose that the most critical targets in a pathogenic organism (e.g. Mtb) should be evolutionarily conserved. Recently, a Genome-wide *Mycobacterium tuberculosis* Variation (GMTV) database was published which catalogues the genome variations in 1623 clinical isolates of Mtb^14^.

In the present work, we have utilized these approaches to identify putative, non-toxic (based on high divergence in human genome) and metabolically critical drug targets, which are evolutionarily conserved and hence should be drug sensitive. Analysis of the 890 targets obtained from the SBSM of the metabolism of Mtb, for their conservation at amino acid level, across the 1623 sequenced genome from its clinical isolates of Mtb (H37Rv as the reference genome), including 1084 Russian MDR and 539 other Mtb strains, using the GMTV database was evaluated. A significant number of genes showed a large number of variations in all the strains. Genes showing no variation in the entire 1623 strains were considered as invariant and could prove to be critical drug targets, based on essentiality and their specific roles in the metabolic pathways. The resulted 33 protein targets with no variation across the reported strains were further evaluated for their existing 3D crystal structures in the Protein Data Bank (PDB). These targets, especially the ones with the available crystal structure can thus be taken up for optimizing inhibitors to generate a drug like compounds library for the development of new leads. Various structure based drug design approaches^15^,^16^,^17^,^18^,^19^,^20^ can be utilized for the development of novel inhibitors for these targets.

We, therefore, propose an integrated methodology that employs systems level analysis, genome-scale variation analysis of the clinical isolates, followed by structure wise selective chemical tailoring of molecules, which has the potential to address the emerging resistance issues as well as fuel the Tb clinical pipeline.

## Results

### Non-Synonymous Variations of the Metabolic Genes in Clinical Isolates

**Figure 1:**
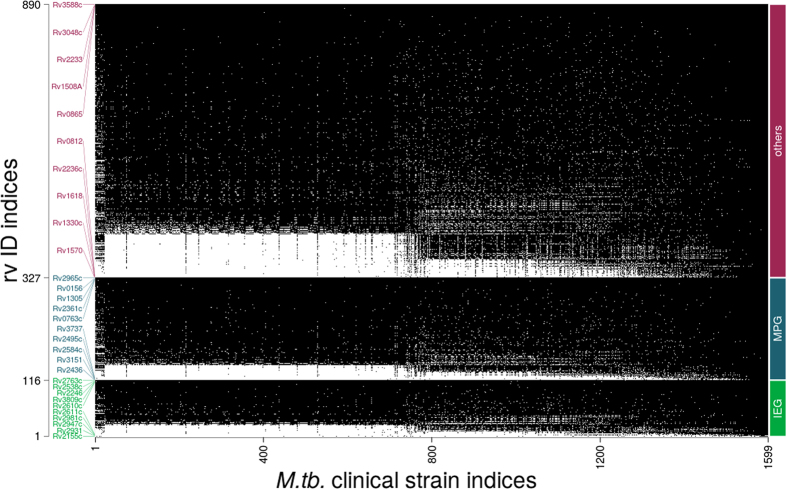
Genes vs genome plot representing non-synonymous variations of 890 metabolic genes, divided into 116 *in silico* Essential Genes (IEG), 211 Metabolic Persister Genes (MPG) and other genes(563) of Mtb in 1623 sequenced clinical isolates (white dots indicate the presence of non-synonymous variation). Least and most mutated 5 genes from each group are shown along the rv ID indices axis.

The 890 Mtb metabolic genes, including 116 *in silico* essential genes (75% of which are experimentally validated) and 211 MPGs, were analyzed for their invariance across the curated genome available from GMTV. The distribution plots of these are shown in Fig. 1.

It was observed that, of the 890 genes, a total of 25 (<3% of the genes, Tables 1 and 2) showed no variation across the 1084 MDR Mtb strains. The resultant invariant genes were mapped back to their corresponding metabolic pathways. These were further analysed based on experimental and *in silico* essentiality and metabolic persister genes. It was interesting to know that 12 of the 25 genes are essential (based on experimental results) and a subset of six of them are MPGs, making them all the more potential targets (Tables 1 and 2). The 25 genes were also evaluated for mutations in the rest of the 539 strains. It was observed that nine of these genes (top nine in Table 1) showed no variations in the remaining 539 strains as well, making them completely invariant genes in 1623 clinical isolates.

In order to rule out the potential sequencing error or sporadic single mutations, genes with single mutations in only one of the 1084 Russian-MDR strains were evaluated. This resulted in an additional subset of 8 new genes (Table 2) having only one non-synonymous variation. Out of the 8, only 3 genes showed experimental essentiality. Thus, in total, these 33 genes were categorized as invariant and can be taken up for their potential as drug targets to MDR Tb. It was observed that, three other genes, *Rv3112, Rv1849* and *Rv2922A* showed one non-synonymous variation in 2, 14 and 40 different Russian-MDR strains, respectively, which were not included in Table 2. Detailed information regarding the number of mutations in various strains for these 36 genes is provided in Supplementary Table 1.

Human mitochondrial genes have an unquestioned bacterial ancestry and therefore, show a high level of conservation with the genes involved in the bacterial respiratory chain. In view of this, 12 genes (out of the set of 33 genes) viz., *Rv2193, Rv3145, Rv0156, Rv1305, Rv3588c, Rv2607, Rv1385, Rv1017c, Rv2465c, Rv0868c, Rv3247c* and *Rv2763c* were found to be involved in the respiration of the bacteria and were rechecked for their conservation across the human mitochondrial proteins. A homology blast analysis was performed to reconfirm that our set of genes has no human homolog, and thus could be safely considered as potential drug targets.

Analysis of the top 15 genes with minimum variation in the GMTV database also revealed some important targets for the purpose of drug discovery. For example, *dfrA, kasB, aroB, rmlA, hisE, pimA, glf, gmk, aroK, mbtC, tesA, ilvE, dapA, dapB, dapE* genes showed minimum number of variations in the genome dataset, out of the 890 metabolic genes, indicating that the current approach of selecting targets based on genome analysis is logical, as most of these genes are highly explored by medicinal chemists. The most important *dapE* gene is involved in the bacterial biosynthesis of lysine and meso-diaminopimelic acid to form diaminoheptanedioate (DAP). Also, as an observation, some of the current and most widely explored targets in the Mtb drug discovery showed high mutations in the genome analysis (e.g. *murG, pks13, ino1, fas, aroG, galTb, tkt, mbtF, galU, pks8, ddl, pks15, ppsA, murD*), making them less important targets to develop the inhibitors, according to the current methodology.

### Structure based analysis of the invariant genes

890 proteins were analyzed for their availability of a PDB structure and it was found that a total of 140 genes/proteins have one or more known PDB structure associated with them. Out of the above 33 set of genes, only 15 proteins had PDB structure available, as indicated in Tables 1 and 2. Seven genes, *Rv3607c, Rv2361c, Rv3048c, Rv2244, Rv1094, Rv3247c* and *Rv2763c*, out of these, were found to be essential based on experimental evidences. Two of the targets, *Rv3607c* and *Rv2361c* were also among the MPGs. These targets can thus be taken up for the structure based drug design approaches.

Although, the 33 drug targets are evolutionarily invariant in the clinical isolates of MDR and non-MDR Tb, we performed the druggability assessment, i.e., the ability of the protein to bind with a drug-like molecule, of these targets for designing inhibitors. The 15 targets, with an available PDB structure were evaluated using TuberQ database^20^. Of these 15 targets, 10 (Rv3048c, Rv3607c, Rv1094, Rv3247c, Rv2763c, Rv2965c, Rv3588c, Rv0865, Rv0321, Rv0098) were highly druggable (DS Index > 0.70–0.90), 3 (Rv2244, Rv0390 and Rv2361c) were druggable (DS Index: 0.53–0.67) and only 2 (Rv2465c and Rv2607) were poorly druggable (DS Index: 0.32–0.44) and none of them were non druggable. It is interesting to note that two poorly druggable targets, although invariant (Rv2607 has 1 non-synonymous variation), were neither *in*-*silico* essential nor experimentally validated essential. It has been observed that all the essential- invariant genes in our study exhibited a high DS index, thus validating our approach of selection of targets, which could be further utilized for the development of small molecule inhibitors.

Additionally, in view of the fact that TuberQ takes into account open reading frame (ORF) sequences and generates structures of unsolved ORFs, we have also carried out the druggability assessment on other 18 targets, for which the PDB structures are unavailable. It was interesting to note that out of 18 targets, 12 generated results, as the rest did not have compatible template structure for modeling. Of these 12 targets, 9 (Rv1202, Rv1436, Rv3053c, Rv1017c, Rv0632c, Rv3600c, Rv3052c, Rv1385, Rv0868c) were highly druggable (DS Index = 0.70–0.97), 2 were druggable (Rv1082, Rv1711; DS = 0.65–0.68) and 1 was poorly druggable (Rv0763c; DS Index = 0.21). There were no non-druggable targets. It was observed that most of the invariant genes showed high DS Index thus validating our approach for selection of potential targets which are evolutionarily conserved.

**Figure 2:**
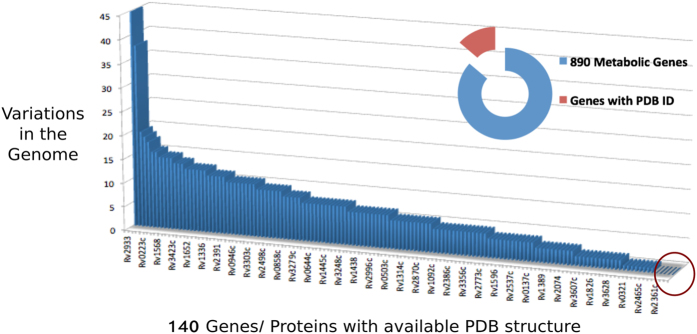
Plot of 890 metabolic genes of Mtb with available PDB structure and the non-synonymous variation data from the 1623 clinical isolates.

A large number of genes out of 140, whose PDB structure of proteins are available, showed significant number variations in the genome. As an example, gene *Rv2933* showed a maximum of 45 variations of a sequence in various strains (Fig. 2 and Supplementary Table 2). Interestingly, *Rv1908c*, gene for *katG*, also showed a large number of variations in the clinical isolates.

## Discussions

In the light of the current situation of drug resistance and tolerance by the bacterium, several analyses have been performed to predict the novel drug targets^11^,^21^,^22^,^23^,^24^,^25^. Most of the analyses involves prioritizing of the metabolic targets based on the uniqueness in metabolome and similarities to known druggable proteins^21^; analyzing protein- protein interactome, flux balance analysis of reactome, experimentally derived phenotypic essentiality data^22^; use of genetic, biochemical and pharmacological data and sequence/structural analysis of targetability using different algorithms^23^, for predicting new targets. Some groups have worked on different aspects involving the assessment of proteins targeted by RNOS in Mtb, based on the fact that the bacteria fights hosts RNOS attack for its survival^24^.

In our previous work on systems level mapping of metabolic complexity in Mtb^11^, we had carried out the functional re-annotation of the *Mtb* genome. The iNJ661^25^ reconstruction was our starting point. Its inconsistencies were removed, and additional gene-reaction associations were incorporated from various databases such as KEGG, Biocyc, MetaCyc, SEED as well as reference textbooks from PubMed, resulting in the iOSDD890, which formed the basis for our present work.

In order to identify the new targets for MDR Tb, we have used the criteria of identifying the invariant genes among the 890 metabolic genes in 1084 genome of MDR Tb. A large number of genes (~200) out of 890 genes showed extensive variation and only a small number of genes were found to be completely invariant. Similarly, about 20% of the 211 MPGs (identified *in silico*), showed extensive non-synonymous mutations in the 1623 clinical isolates. Only 7 of these MPGs were found to be invariant. The functions of the 33 invariant genes, as well as the involvement in various metabolic pathways are shown in Tables 1 and 2. A number of these invariant genes also have well characterized 3D protein structures, as shown in Tables 1 and 2, which is important for the development of a novel inhibitor. It was interesting to note that out of 140 metabolic proteins/genes having the PDB structures; only 15 were invariant, whereas, some of the genes showed extensive mutations all along the polypeptide chain in the 1623 clinical isolates. Genes viz., *Rv1305, Rv2361c, Rv1202, Rv2193*, and *Rv1436* have shown least number of mutations in the 1623 clinical isolates and some are also experimentally essential, which could emerge out to be highly potential drug targets. *Rv1305* or *atpE* is a lipid binding protein involved as one of the three chains of the non-enzymatic component (cf(0) subunit) of the ATPase complex (KEGG Pathway ID: mtu00190, mtu01100)^26^,^27^,^28^. *Rv2361c* is involved in the synthesis of decaprenyl diphosphate, a molecule having a central role in the biosynthesis of most features of the mycobacterial cell wall (KEGG pathway ID: mtu00900)^29^. These two are also a part of MPGs, which appear on the emergence of persistent phenotype upon drug pressure, thereby resulting into major drug tolerance in the bacteria. Gene *Rv1202* or *dapE* is another essential gene, involved in the dap pathway for the biosynthesis of lysine (KEGG pathway ID: mtu00300)^30^,^31^,^32^. The latter is a very well explored pathway for the design of novel antibiotics. Lysine and meso-DAP are vital constituents of the bacterial cell wall^32^,^33^. Also, mammals lack the ability to biosynthesize lysine, therefore, the occurrence of lysine biosynthesis pathway in only microorganisms and plants make it an ideal target for the designing of novel antibacterial agents with low mammalian toxicity^34^. *Rv2193* or *ctaE* (Cytochrome C Oxidase, subunit III) is known to be involved in aerobic respiration in the bacteria (KEGG pathway ID: mtu00190, mtu01100)^35^,^36^,^37^ and is a metabolic persister gene as well as an experimentally essential one. Gene *Rv3145* or *nuoA* is NADH dehydrogenase I (Chain A), involved in aerobic respiration of the bacteria (KEGG pathway ID: mtu00190, mtu01100)^28^. From our previous analysis, the two genes (*Rv2193* and *Rv3145*) are also a part of MPGs^11^.

**Figure 3:**
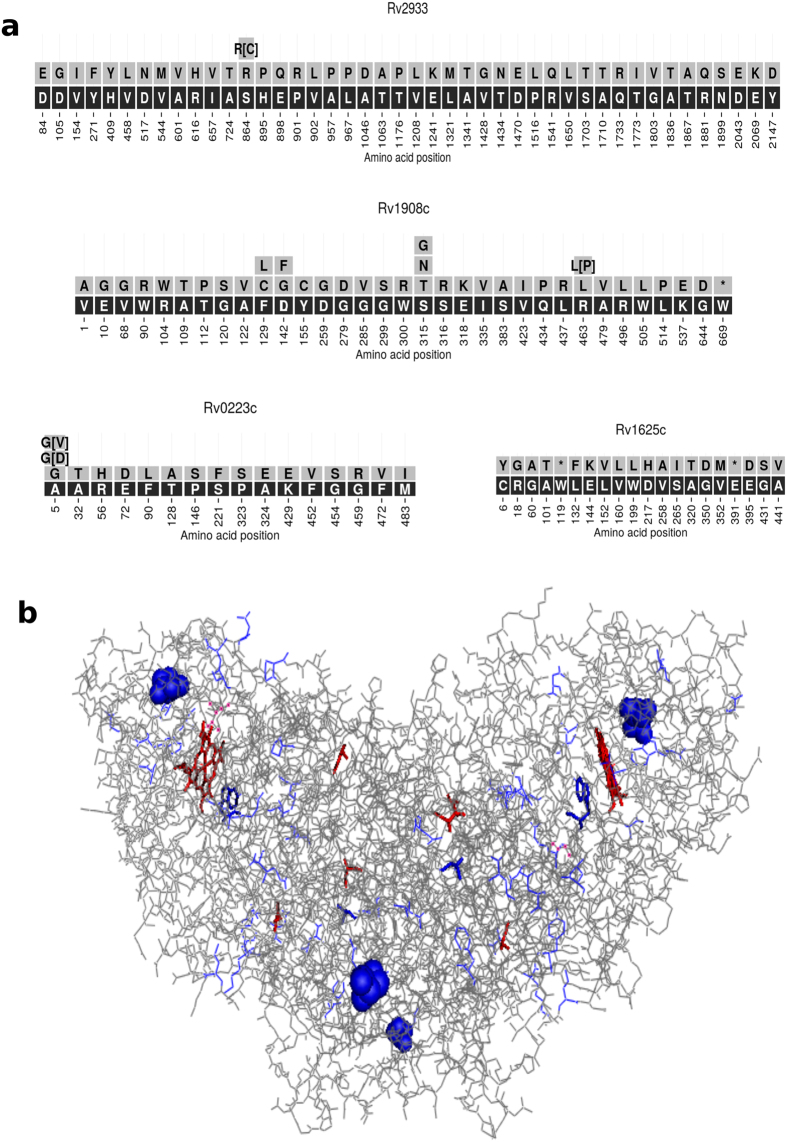
(**a**) Mutation data of the top four variant genes out of the 140 genes with available PDB structure; (**b**) wired model of the protein 1SJ2, *Rv1908c* (katG), which is a homo-2-mer, showing the major sites of mutation in the binding pocket (Mutations marked in blue; ligand HEM and co-solvent Glycerol, GOL marked in red).

The implication of extensive genomic variations leading to the MDR was also explored and it was observed that *Rv2933, Rv1908c, Rv0223c, Rv2447c* and *Rv1625c* showed maximum number of variations out of 140 genes and were further evaluated for the sites of these variations in the protein structures (Fig. 3a). Some of the mutations were observed in the binding pockets of the proteins, e.g. *Rv1908c* (PDB ID: 1SJ2, Gene Name: *katG*) (Fig. 3b). The *Rv1908c* gene is known to catalyze Isoniazid (INH) for its pharmacological activity. Mutations in the same may result in the drug resistance of the bacteria towards INH. Hence, understanding these mutations in the protein structure of *katG* becomes imperative.

**Figure 4:**
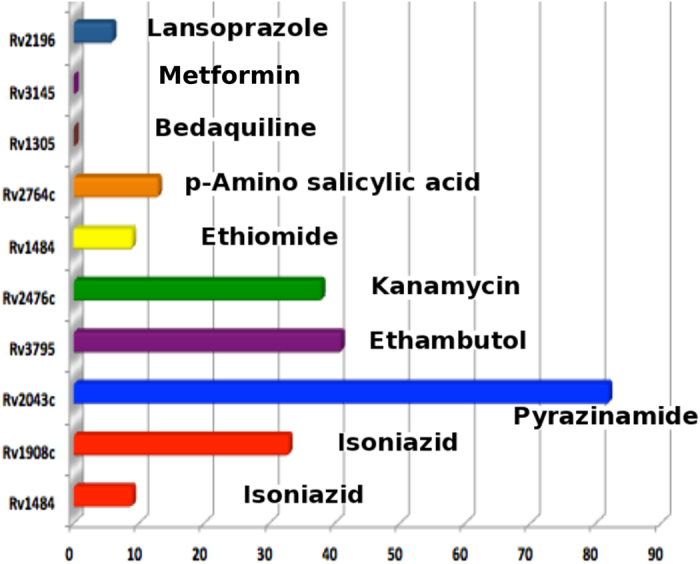
Plot of number of mutations vs the targets of the known antibiotics and the name of the corresponding drug.

In order to understand the drug resistance, we have also evaluated the 890 genes as important targets of the known drugs (first and second line of Tb drug regime), which includes the most common antibiotics viz., Isoniazid, Pyrazinamide, Ethiomide, etc; for their number of variations. The analysis showed that the current set of targets show a high degree of mutations in the bacterial genome. Some of the lowest variations in the data set were observed for targets, which are yet unexplored or have a recent new inhibitor designed for them (Fig. 4). Although, *Rv1908c* or *katG* is highly mutated, the other target *Rv1484* of isoniazid, showed relatively lower variations compared to other known drug targets. Also, *atpE (Rv1305*), target for a recent drug molecule for MDR Tb, Bedaquiline^38^, showed no variation in the entire 1623 Mtb strains, supporting the hypothesis of the importance of completely invariant genes as potential targets for the successful development of novel antibiotics. Also, from the point of view of repurposing of the existing drugs, analysis of the recent report on Lansoprazole^39^, a well known drug for heart burn (novel class of cytochrome bc1 inhibitor), has shown a good *in vitro* and *in vivo* activity against Mtb. According to our analysis, the target for Lansoprazole, *Rv2196*, shows very few mutations in the entire Mtb strains, further strengthening the hypothesis. Metformin, a popular drug for type-II diabetes has also been recently proposed to inhibit bacterial NDH- I, which is encoded by *nuoA (Rv3145* in Mtb)^40^, thereby opening possibilities of adjunct therapy for tuberculosis. The gene shows no mutation in 1084 Russian MDR strains and is also a part of MPGs. The three targets viz., *Rv2193, Rv2361c* and *Rv1305* (Table 1) showing least number of variations are also a part of the MPGs, and point towards the fact that these can actually form a new set of targets to develop 2^nd^ line-Tb drug regime for MDR and XDR patients. Also, *Rv2196*, the genes targeted by Lansoprazole was analysed for variations in the Russian MDR strains, which showed 4 different non-synonymous variations in 9 different strains of MDR-Tb. *Rv3145* for Metformin, shows two different types of mutations in three different clinical strains of MDR-Tb, indicating the possibilities of drug repurposing for MDR Tb. Details of the non-synonymous mutations in the targets of these known drugs (Fig. 4) in the MDR clinical isolates along with the sites of mutations in their protein structures (wherever the PDB structure is available) were also evaluated. The knowledge of the mutation sites in the protein structure of the existing drug targets was considered crucial for understanding the origin of drug resistance and for the availability of any other binding site for drugs design.

The lower the concentration of a target protein/enzyme for non-competitive inhibitors or a tight binder (most of the known antibacterials are non-competitive inhibitors), the lower will be the antibiotic’s minimum inhibitory concentration (MIC) and hence, the more its efficacy. In this context, we had analysed the intracellular levels of all the proteins coded by the 33 invariant genes based on the complete proteome of Mtb, as reported recently^11^. We also found that, most of these 33 targets are highly druggable as presented above.

In order to search for a new Mtb drug candidate and fulfill the challenging task of designing new chemical entities which are in accordance with the Lipinski’s Rule, we can undertake a detailed chemical analysis of the existing drug pipeline for finding out the important building scaffolds possessed by the highly active molecules. The selected invariant targets can thus be analysed for the presence of a known inhibitor in various databases to initiate the process of ligand designing.

Recent study on the construction of a synthetic organism with minimal genome revealed existence of a class of ‘quasi-essential’ genes which are required for the optimal growth of the organism^41^. This indicates that even some non-essential genes in the organisms, which are involved in important pathways, can be crucial for the survival, hence understanding of the sites of mutations in the genes becomes a viable approach for generating new targets for drug discovery. Our systems level analysis of the metabolic genes of Mtb, which incorporates an extensive genome wide evaluation, including the understanding of the sites of mutations in the clinical isolates to identify the highly potential non-toxic targets, followed by a proteome based analysis to design and generate ligands, has the potential of fuelling the drug discovery pipeline for the generation of new leads for Mtb.

Therefore, we propose that this comprehensive integrated methodology, with both experimental and *in silico* approach has the potential to not only tackle the MDR form of Mtb but also the most important persister population of the bacterium. Our analysis for the prediction of drug targets is based on the reference to a wealth of literature information, providing evidences from both *in vitro* and *in vivo* experimental results.

## Methods

The present study uses the list of 890 metabolic genes in Mtb, reported in our previous SBSM model^11^, to evaluate their conservation across the available annotated Mtb genome, thereby exploiting the most critical and highly invariant genes as potential drug targets.

### Determination of the invariant genes across the 1623 Mtb Genome: gene variation distribution plot

**Figure 5:**
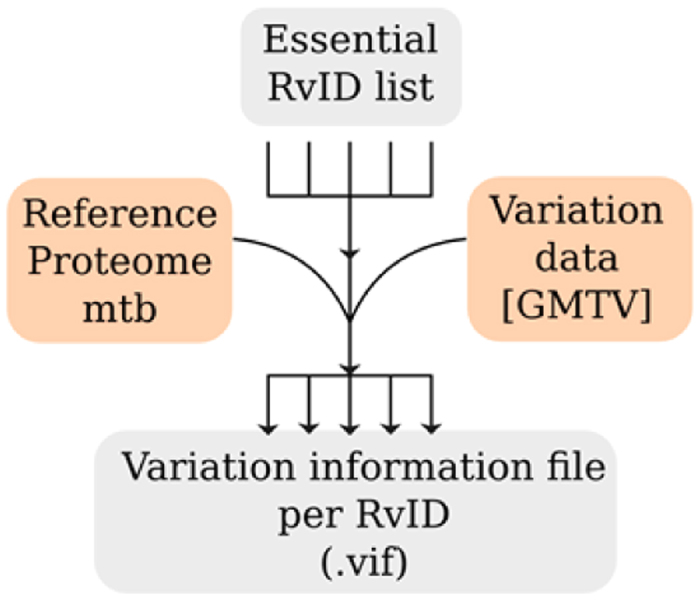
Flow chart representation of the invariant genes analysis.

Variation data of the Mtb clinical isolates was downloaded from GMTV database (http://mtb.dobzhanskycenter.org/). A total of 1623 isolates had a unique strain from a particular origin, which includes a 1084 MDR strains from the Russian origin. The data was used to determine the invariant proteins after applying a quality threshold of 30 (probability of incorrect base is 1 in 1000), which included all the 1084 MDR strains. The criterion for invariance is proteins with maximum one variation in 1084 Russian MDR strains and less than 5% in the total strains (1623) at any site. Also, only one variation in one strain, out of 1084 Russian-MDR strains was considered in order to rule out some sequencing errors. At first, protein FASTA file of reference genome from NCBI (NC000962.3) and the variation of all the genome sequence reported in GMTV database, were downloaded. The gene sequence variations were computationally translated and only non-synonymous amino acid variations were used for further analysis as depicted in Fig. 5. Thus, for all the RvIDs, we have generated variation information file (.vif) that contains variation across 1623 strains of Mtb.

Using vif files for each RvID, protein FASTA files were generated, where all non-synonymous variations in the Mtb isolates were stored through mapping positions with respect to the reference protein sequence. We have excluded strains for which the reported reference amino acid or its position was conflicting with the reference sequence downloaded. For each RvID, a figure was generated which represents the amino acid seqeunce of a particular gene and the variations that occur in it. As an example, Fig. 3(a) represents the variation in the amino acid sequence and their frequency in clinical isolates for top 4 most variant genes, for which the PDB structures were available. Dataset used in our analysis is available online at https://rintukutum.github.io/invariant-genes-mtb/.

Statistical signicance was performed using non-parametric statistical test, Wilcoxon rank sum test, between IEG and others (p-value = 0.185) IEG and MPG (p-value = 0.112) others and MPG (p-value = 0.442). The alpha value was set for 0.05 for the analysis.

## Additional Information

**How to cite this article:** Kaur, D. *et al*. Data Intensive Genome Level Analysis for Identifying Novel, Non-Toxic Drug Targets for Multi Drug Resistant *Mycobacterium tuberculosis. Sci. Rep.* **7**, 46595; doi: 10.1038/srep46595 (2017).

**Publisher's note:** Springer Nature remains neutral with regard to jurisdictional claims in published maps and institutional affiliations.

## Supplementary Material

Supplementary Information

Supplementary Table 1

Supplementary Table 2

## Figures and Tables

**Table 1:** List of Invariant Genes (1–9- No variation in the entire 1623 strains, 10–25- No variations in 1084 Russian MDR strains), from 1623 clinical isolates out of 890 metabolic genes.

RvID	Gene Name	Function	PDB ID
*Rv0156*	*pntAb*	NAD(P) transhydrogenase (subunit alpha)	No
*Rv0763c*	*Rv0763c*	Ferredoxin	No
*Rv1305*^#,*,⌘^	*atpE*	F0F1 ATP synthase subunit C	No
*Rv2361c*^#,*^	*Rv2361c*	Z-decaprenyl diphosphate synthase	2VG2, 2VG3, 2VG4
*Rv2965c*	*kdtB*	Phosphopantetheine adenylyltransferase	1TFU, 3PNB, 3LCJ, 3NBA, 3NBK
*Rv3588c*	*canB*	Catalyzes reversible dehydration of CO2 to form bicarbonate	1YM3, 2A5V
*Rv3048c*^#^	*nrdF2*	Involved in the DNA replication pathway	1UZR
*Rv1508A*	*Rv1508A*	Function unknown	No
*Rv0865*	*mog*	Involved in molybdopterin biosynthesis	2G4R
*Rv0321*	*dcd*	Interconversion of dCTP and dUTP	2QLP, 2QXX
*Rv0632c*	*echA3*	Enoyl-CoA hydratase	No
Rv1082^*^	*mca*	Mycothiol conjugate amidase	No
*Rv1202*^#,^^	*dapE*	Dipeptidase	No
*Rv1436*^#,*^	*gap*	Glyceraldehyde-3-phosphate dehydrogenase	No
*Rv2193*^#,*^	*ctaE*	Cytochrome C oxidase subunit III	No
Rv3145^*,❖^	*nuoA*	NADH dehydrogenase I (chain A)	No
*Rv3600c*	*Rv3600c*	Pantothenate kinase	No
*Rv3607c*^#,*^	*folB*	Dihydroneopterin aldolase	1NBU
*Rv3052c*	*nrdI*	Probably involved in ribonucleotide reductase function.	No
*Rv3053c*^#^	*nrdH*	Involved in electron transfer system for ribonucleotide reductase system NRDEF	No
*Rv2244*^#^	*acpM*	Involved in fatty acid biosynthesis (mycolic acids synthesis)	1KLP
*Rv1017c*^#^	*prsA*	Probable ribose-phosphate pyrophosphokinase PrsA	No
*Rv2465c*	*rpiB*	Interconverts ribose-5-phosphate and ribulose-5-phosphate	1USL, 2BES, 2BET, 2VVO, 2VVP, 2VVQ
*Rv1711*^#^	*Rv1711*	RNA pseudouridine synthase	No
*Rv3374*	*echA18.1*	enoyl-CoA hydratase activity	No

**Table 2:** List of Genes with one non-synonymous variation in only one of the Russian strains.

RvID	Gene Name	Function	PDB ID
*Rv2607*	*pdxH*	Involved in biosynthesis of pyridoxine (vitamin B6) and pyridoxal phosphate	2A2J
*Rv0098*	*fcoT*	Involved in fatty acid metabolism	2PFC
*Rv0390*	*Rv0390*	Function unknown	2FSX
*Rv1385*	*pyrF*	Involved in the biosynthesis of pyrimidines	No
*Rv0868c*	*moaD2*	Involved in molybdenum cofactor biosynthesis.	No
*Rv1094*^#^	*desA2*	Conversion of saturated fatty acids to unsaturated fatty acids	1ZA0
*Rv3247c*^#^	*tmk*	Probable Thymidylate Kinase TMK (dTMP Kinase) (Thymidilic Acid Kinase) (TMPK)	1G3U, 1GSI, 1GTV, 1MRN, 1MRS, 1N5I, 1N5J, 1N5K, 1N5L, 1W2G, 1W2H
*Rv2763c*^#^^	*dfrA*	Essential step for *de novo* glycine and purine synthesis & dihydrofolate reductase activity	1DF7, 1DG5, 1DG7, 1DG8, 2CIG

^#^Essentiality based on experimental results; ^**^**^Essentiality based on *in silico* analysis, ^*^Metabolic Persister Genes; Proposed target for Metformin; ^⌘^Target for Bedaquiline; (a recent drug molecule).
