# Erratum: Data Intensive Genome Level Analysis for Identifying Novel, Non-Toxic Drug Targets for Multi Drug Resistant *Mycobacterium tuberculosis*

**DOI:** 10.1038/srep46825

**Published:** 2017-07-07

**Authors:** Divneet Kaur, Rintu Kutum, Debasis Dash, Samir K. Brahmachari

Scientific Reports 7: Article number: 4659510.1038/srep46595; published online: 04 20 2017; updated: 07 07 2017

This Article contains a publication error in the legend of Table 2.

“^#^Essentiality based on experimental results; ^Essentiality based on *in silico* analysis; *Metabolic Persister Genes; Proposed target for Metformin; ^⌘^Target for Bedaquiline; (a recent drug molecule)”.

should read:

“^#^Essentiality based on experimental results; ^Essentiality based on *in silico* analysis; *Metabolic Persister Genes; ^❖^Proposed target for Metformin; ^⌘^Target for Bedaquiline; (a recent drug molecule)”.

