# Supplementary material for: Data Intensive Genome Level Analysis for Identifying Novel, Non-Toxic Drug Targets for Multi Drug Resistant Mycobacterium tuberculosis
Source: Sci Rep. 2017 Apr 20;7:46595. doi: 10.1038/srep46595 (PMC5397868; doi:10.1038/srep46595)
Supplement: Supplementary Information [file srep46595-s1.doc]

**Data Intensive Genome Level Analysis for Identifying Novel, Non-Toxic Drug Targets for Multi Drug Resistant *Mycobacterium tuberculosis***

Divneet Kaur^1^, Rintu Kutum^1,2^, Debashish Dash^1,2^ & Samir K. Brahmachari^1,2,3^*

^1^CSIR- Institute of Genomics and Integrative Biology, New Delhi, India

^2^Academy of Scientific and Innovative Research, New Delhi, India

^3^CSIR- Open Source Drug Discovery Unit, New Delhi, India

**Supplementary Information provided in the spreadsheet files:**

Supplementary Table 1: Set of invariant genes (with a maximum of one variation in <5% of 1084 Russian MDR- Strains), with exact number of variations in exact number of strains.

Supplementary Table 2: List of 140 genes with existing PDB structure and number of non-synonymous mutations.
